# Report of a Case of Nephrolithotomy*Reported to the Chicago Medical Society, November 21, 1887.

**Published:** 1887-12

**Authors:** W. T. Belfield

**Affiliations:** Surgeon to the Cook County and the Maurice Porter Hospitals


### REPORT OF A CASE OF NEPHROLITHOTOMY.*

BY W. T. BELFIELD, M. D.,

SURGEON TO THE COOK COUNTY AND THE MAURICE PORTER HOSPITALS.

James F——, thirty-seven years old, gives the following history: Some fifteen years ago he suffered for several months from great irritability of the bladder, urinating very frequently and with considerable pain. On several occasions he passed blood. He was treated at that time for "cystitis." These symptoms gradually disappeared. At intervals thereafter he suffered from dull pain in the loins, often extending upward to the chest, and sometimes incapacitating him for work. For the past two years pus had been seen in the urine, constantly, in varying quantity. At times the amount would be small for a few days, during which time the pain in the side became severe; then a large quantity of pus would be discharged while the pain diminished. After treatment for various complaints he came, about a year ago, under the care of Dr. Frank Billings, who discovered an increased area of dullness over the left kidney, and at times a distinct though slight swelling in the loin. Recognizing that the left kidney was the seat of suppuration, Dr. Billings endeavored to ascertain the cause thereof. Tuberculosis was almost certainly excluded by repeated examinations of the pus for the characteristic bacilli, with negative result. As renal calculus seemed the most probable diagnosis, Dr. Billings referred the patient to me for operation. After thorough examination and observation this seemed to me also the most plausible diagnosis, though many of the acute symptoms of calculus did not then exist. The right kidney could be felt through the abdominal walls and seemed normal; yet, previous to the operation, I endeavored to demonstrate its integrity by closing the ureter of the one under suspicion, by means of Silbemann's catheter. The instrument caused so much pain, however, that the result was unsatisfactory. June 26, last, in the presence of Drs. Billings, Hosmer and Tilley, I explored the left kidney through an oblique lumbar incision. The organ was found to be enlarged and flabby. Upon incising the cortex an explanation of this condition was found in a slight hydro-nephrosis. The cause of the hydro-nephrosis was found in a calculus of conical shape, some two inches in length, its smaller end impacted in the ureter so tightly as to require some force for its extraction. A few loose flakes were afterwards removed from the pelvis and ureter. Recovery ensued without notable event. On the 14th day the patient visited his place of business; in two months his weight increased from 105 to 123 pounds, and all morbid symptoms had disappeared, except that some pus was still present in the urine; this

* Reported to the Chicago Medical Society, November 21, 1887.

has now become so slight in amount as to be discoverable only with the microscope. Presumably the shriveling of the distended kidney is about accomplished. The stone is an oxalate or mulberry calculus, and weighed 244 grains.

